# Dielectric Characterization of Core-Shell Structured Poly(vinylidene fluoride)-*grafted*-BaTiO_3_ Nanocomposites

**DOI:** 10.3390/polym15030595

**Published:** 2023-01-24

**Authors:** Fatima Ezzahra Bouharras, Massimiliano Labardi, Elpidio Tombari, Simone Capaccioli, Mustapha Raihane, Bruno Améduri

**Affiliations:** 1 IMED-Lab, Faculty of Sciences and Techniques, Cadi-Ayyad University, Av. Abdelkrim Khattabi, BP 549, Marrakesh 40000, Morocco; 2 Physics Department, University of Pisa, Largo Pontecorvo 3, 56127 Pisa, Italy; 3 ICGM, University of Montpellier, CNRS, ENSCM, 34095 Montpellier, France; 4 CNR-IPCF, Pisa Unit, Physics Department, University of Pisa, Largo Pontecorvo 3, 56127 Pisa, Italy; 5 CISUP, Centro per l’Integrazione della Strumentazione dell’Università di Pisa, 56126 Pisa, Italy

**Keywords:** broadband dielectric spectroscopy, barium titanate nanoparticle, Poly(vinylidene fluoride), core-shell, RAFT polymerization, interfacial polarization

## Abstract

Dielectric properties of poly(vinylidene fluoride)-*grafted*-BaTiO_3_ (PVDF-*g*-BT) core-shell structured nanocomposites obtained from Reversible Addition Fragmentation chain Transfer (RAFT) polymerization of VDF were investigated by Broadband Dielectric Spectroscopy (BDS). The dielectric constant increased along with the BT content, about +50% by addition of 15 vol% of BT, which was around 40% more than expected from predictions using the usual dielectric modeling methods for composite materials, to be ascribed to the effect of the interfacial core-shell structure. The known dielectric relaxations for PVDF were observed for the neat polymer as well as for its nanocomposites, not affected by the presence of nanoparticles. A relaxation process at higher temperatures was found, due to interfacial polarization at the amorphous-crystalline interface, due to the high crystallinity of materials produced by RAFT. Isochronal BDS spectra were exploited to detect the primary relaxation of the amorphous fraction. Thermal analysis demonstrated a very broad endotherm at temperatures much lower than the usual melting peaks, possibly due to the ungrafted fraction of the polymer that is more easily removable by repeated washing of the pristine material with acetone.

## 1. Introduction

In the recent years, significant developments in energy storage systems have been reported due to their scientific as well as technological importance [1]. Among them, dielectric nanocomposites have drawn great attention because of their wide range of use [2,3,4].

At present, the synthesis of a dielectric nanocomposite by the introduction of high dielectric constant inorganic nanoparticles into an organic polymer matrix is a promising way to obtain high energy density materials. In general, the energy density *U*_e_ can be expressed as follows:

*U*_e_ = 1/2 *ε*_0_ *ε*_r_ *E*_b_^2^, (1)

where *ε*_0_ is the permittivity of free space (8.854 × 10^−12^ F/m), *ε*_r_ the relative permittivity, and *E*_b_ the dielectric breakdown field. These materials properties are of highest interest during the preparation of a dielectric nanocomposite. Thus, high energy densities could be reached by either increasing dielectric permittivity and/or dielectric breakdown. Moreover, the nanocomposite structure should have a balance of both nanofillers and polymer properties to lead to an increase in energy storage.

Poly(vinylidene fluoride) (PVDF), as well as its copolymers, has been widely reported in the literature due to its attractive ferroelectric properties [5,6]. PVDF is a semicrystalline polymer that presents glass transition temperature *T*_g_ in the range of −40 to −30 °C, melting temperature between 155 and 192 °C, and Curie temperatures between 195 and 197 °C [5]. This fluorinated hydrocarbon polymer has a repeat unit (-CH_2_CF_2_-) that can exhibit different crystalline polymorphs, named α, β, γ, δ and ε [7]. The β phase is the one with the highest permittivity, as well as thermodynamically most stable (melting enthalpy of 219.7 J/g [8]) than the α phase (melting enthalpy of 104.7 J/g) that on the other hand, is the most probable to be formed, for crystallization kinetics reasons. Both α and β crystalline polymorphs phases have about the same melting temperature of 167–172 °C.

The dielectric constant of PVDF lies in the range of 6 to 12 [9]. By introducing high dielectric constant nanofillers into the PVDF matrix, such a value can be improved [10,11,12,13,14,15,16]. Barium titanate (BaTiO_3_, abbreviated as BT) is one of the most explored perovskite ferroelectric materials, thanks to its attractive dielectric and piezoelectric properties, leading to its wide employment in several applications such as in electronic industry [17,18,19,20,21,22]. BT generally exhibits high dielectric permittivity that can be affected by the nanoparticle size. Mao et al. [23] investigated the effect of nanoparticle size on dielectric properties of PVDF/BT nanocomposites. It was found that the highest permittivity of nanocomposites was obtained for nanoparticle size around 80–100 nm, reaching a maximum value of 93 (at 1 kHz) for 100 nm size. However, the dielectric permittivity remained nearly constant (~65 at 1 kHz) for particle size larger than 250 nm, and decreased down to ~48 with particle size of 50 nm.

Another important factor that affects dielectric permittivity of nanocomposites is the interface between ceramic nanoparticles and polymer matrix [24,25]. Thus, the selection of an appropriate pre-treatment of nanoparticles may be crucial to obtain high energy storage nanocomposites. Dang et al. [26] used a commercial silane agent (KH550: HN_2_(CH_2_)_2_Si(OC_2_H_5_)_3_) to functionalize the BT nanoparticle surface and improve its compatibility with the PVDF matrix. To be able to observe the effect of the modifier agent, different KH550 amounts were used. An optimum concentration of 1.0 wt% was found to give the highest permittivity values, which increased the composite permittivity from 45 to 52 (+16%) at 1 kHz for 700 nm diameter particles. Dalle Vacche et al. [14] obtained an increase of permittivity by 98% (at 0.5 kHz) by adding 15 vol% of 200 nm BT nanoparticles to PVDF-TrFE; however, such an increase was reduced by surface modification of BT by silanes. Carbone et al. [15] obtained even higher permittivity enhancements (42 at 1 kHz vs. the nominal permittivity of 12 for PVDF-TrFE) with 30 vol% loading of 1 μm BT nanoparticles, that could be further enhanced by surface modification. Horchidan et al. [16] obtained +56% permittivity enhancement at 1 kHz with 20 vol% loading of 60 nm BT nanoparticles to PVDF, further enhanced by surface modification with Ag. As a comparison, we could obtain +49% enhancement at 0.5 kHz by adding PVDF with 15 vol% of 100 nm BT nanoparticles with grafted PVDF surface modification, as shown in this work.

The main goal of this work deals with extensive dielectric characterizations performed on nanocomposites consisting on BaTiO_3_ nanoparticles as fillers and PVDF as the polymer matrix, prepared by means of Reversible Addition Fragmentation chain Transfer agent (RAFT) polymerization, with a grafting-from procedure in order to obtain BaTiO_3_ nanoparticles with grafted PVDF chains, in a core-shell structure. In particular, Broadband Dielectric Spectroscopy (BDS) was applied to the as-prepared nanocomposites, in the form of pressed pellets, to provide their dielectric permittivity as a function of frequency. BDS is also able to detect dielectric relaxation processes, such as the primary (or *α*) relaxation, due to segmental motion of the polymer chains, as well as secondary (*β*) processes due to relaxation of molecular units. Furthermore, processes related to the phase-segregated structure in semicrystalline polymers or nanocomposites, due to relaxation of mobile charge carriers accumulated at the interfaces, referred to as Maxwell-Wagner-Sillars (MWS) relaxations, can be also demonstrated. Therefore, BDS can provide information on the glass transition (temperature and dynamic fragility), the presence of mobile charge carriers, as well as on the structure of the semicrystalline material with or without inorganic nanoinclusions. The effect of BT loading on dielectric permittivity was also studied, by comparing nanocomposites with different amounts of filler. Special attention was devoted to the effect of the interface related to the core-shell structure of our grafted nanoparticles. In addition, BDS data were complemented by Differential Scanning Calorimetry (DSC) thermograms, to better elucidate the peculiar phase structure of RAFT-produced PVDF.

## 2. Materials and Methods

### 2.1. Materials

PVDF-*grafted*-BaTiO_3_ (abbreviated as PVDF-*g*-BT) nanocomposites produced with different loadings of BaTiO_3_ were studied. Synthesis of this kind of nanocomposites is reported elsewhere [27].

The barium titanate nanoparticles were obtained commercially. The specifications from the company report: BaTiO_3_, 99%, Aldrich CAS: 12047-27-7 nanopowder (cubic crystalline phase), particle size: <100 nm (BET), dielectric constant 150. TEM characterization of barium titanate nanoparticles after PVDF grafting reaction was reported in a previous work (Ref. [27]), where the shape and size of nanoparticles could be evaluated, as well as the presence of grafted polymer on their surface. Additionally, XRD characterization of barium titanate nanoparticles has been reported in the same work (Ref. [27]), where the XRD patterns of the as-received nanoparticles exhibited characteristic crystallographic peaks at 22°, 31°, 38°, and 45°, assigned to the diffraction planes (100), (110), (111) and (200), respectively, and corresponding to the cubic phase of BaTiO_3_.

The as-received BaTiO_3_ nanoparticles were surface-modified by anchoring xanthate functions to their surface. Then, grafting of PVDF onto the functionalized surface was performed by RAFT polymerization of VDF. Purification by repeated washing with acetone was performed to remove physisorbed (ungrafted) polymer from the nanocomposite. All samples were annealed at 60 °C until residual solvent was removed. The ungrafted polymer extracted from one of the nanocomposites was also considered in our study. To compare the effect of the addition of BT nanofiller to the PVDF polymer matrix, PVDF was also synthesized by RAFT polymerization of VDF in the presence of potassium ethyl xanthate and initiated by *tert*-butyl peroxypivalate (RAFT PVDF). SEM-EDX analysis of nanocomposites, confirming the presence of BaTiO_3_ and PVDF, was also reported in Ref. [27]. The mechanism for RAFT grafting of BT onto PVDF was also described in the same Ref. [27].

Finally, a commercial, high molar mass PVDF (Solef^®^ 1010, Solvay, Brussels; M_w_ = 352 kDa, dispersity *Đ* = 2.3) was also investigated for comparison.

### 2.2. Thermal Characterization

Thermogravimetric Analysis (TGA) was performed by a SII TG/DTA 7200 EXSTAR Seiko analyzer (Seiko, Chiba, Japan), under heating from 30 °C to 800 °C, at 10 °C/min rate. Air was fluxed at 200 mL/min during all measurements.

Differential Scanning Calorimetry (DSC) was performed by a Perkin-Elmer DSC 8500 instrument, equipped with intracooler III assembly as refrigeration system. First heating ramps were performed from −80 °C to above the melting point (200 °C) at a rate of 10 °C /min, followed by cooling at 10 °C/min, 20 °C/min, or 200 °C/min down to −80 °C, and a second heating at 10 °C/min, for demonstrating the difference between the as-produced material and the same material after a melting-cooling cycle, leading to a different recrystallization process depending on the used cooling rate.

**Figure 1:**
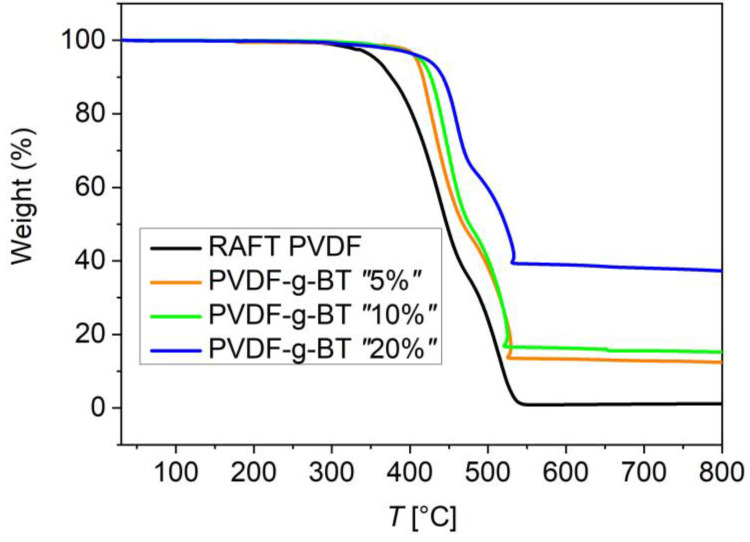
TGA thermograms of the neat RAFT PVDF sample as well as of the nanocomposites with the three different BT fractions.

The TGA thermograms for the four investigated samples are reported in Figure 1. Table 1 shows the weight losses of the obtained nanocomposites at 700 °C obtained by TGA. Actual BT weight and volume fractions could be inferred from these measurements, as also reported in Table 1. We remark that TGA was intentionally performed only under air in order to study the thermal stability of these polymer and composite materials under thermo-oxidative atmosphere, as it is corresponding to the real conditions of their application. These conditions are harsh ones in order to check the suitability of these materials, since fluorinated polymers are usually tested under such oxidative atmospheres. Additionally, since it was necessary to determine the mass fraction of the inorganic component, we need to completely eliminate the organic fraction at the highest temperatures that was accomplished by full oxidation due to the presence of ambient oxygen.

The crystalline fractions of as-prepared samples were obtained by comparing their specific melting enthalpy, measured during the first heating ramp by DSC (Table 1), with that of a 100% α-phase crystalline PVDF, known from the literature [28]. In more detail, specific melting enthalpy Δ*H*_m_, as customary in DSC analysis, was derived by integration of the measured heat flow, after subtraction of a baseline due to the specific heat of both polymer and filler, and then divided by the polymer mass. To obtain the mass of the polymer, the mass fraction of BT was subtracted from the total mass of the nanocomposites. Crystallinity was obtained as *X* = Δ*H*_m_/Δ*H*_c_, where Δ*H*_c_ (104.5 J/g) is the specific melting enthalpy of 100% crystalline α-phase PVDF [28].

Crystallinity rate values resulted around 80% for neat PVDF samples. Such value is higher than the one reported in the literature for PVDF produced by standard methods that is around 35–70% [5]. For the nanocomposites, instead, crystallinity ranges between 89% and 98% (DSC thermograms are reported in Figure S1 of the Supplementary Materials). This could be due to the process of removal of ungrafted polymer (purification), based on repeated washing with acetone, likely being less effective to solve the crystalline phase compared to the amorphous one.

### 2.3. Pellets Preparation

Pellets were prepared with a manual, uniaxial hydraulic press. The sample, in the form of powder, was placed in the compression cylinder. Then, the hydraulic press enabled applying a pressure of 1.25 kBar to the sample through the press piston, for about 30 s. Pellets were also prepared for the as-received BT nanoparticles, as well as for the commercial, high molar mass PVDF powder (Solef^®^), for comparison measurements.

### 2.4. Dielectric Characterization

Broadband Dielectric Spectroscopy (BDS) was performed by an Alpha Analyzer spectrometer by Novocontrol technologies GmbH & Co. (Montabaur, Germany), equipped with a Novocontrol Quatro nitrogen gas flow cryostat. The prepared pellets (10 mm diameter, thickness values of around 400 μm), sandwiched between two thin layers of Pb for more uniform contact with electrodes, were placed in the BDS measurement cell. Isothermal spectra were obtained by recording the dielectric response while ramping the frequency *ω* of the applied sinusoidal voltage in the range from ~5 × 10^−2^ Hz to ~2 × 10^6^ Hz, with logarithmic increments, at constant temperature, held for about 30 min. The explored temperature range was from −100 °C to 125 °C, with intervals of 5 or 10 °C. The amplitude of the applied AC potential was 1.5 V. Isochronal spectra were obtained by ramping temperature in the range from −100 °C to 140 °C, and recording the dielectric response at three fixed frequencies: 10 Hz, 300 Hz, and 10 kHz, while adopting the typical heating and cooling rate used in DSC measurements (10 °C/min). Results are presented in terms of the complex dielectric function ε^*^(*ω*) = *ε*′(*ω*) − *i ε*″(*ω*), where *ε*′ is the real part of the dielectric constant, or permittivity, while *ε*″ is the imaginary part, or dielectric loss.

## 3. Results

### 3.1. BaTiO_3_ Nanoparticles

**Figure 2:**
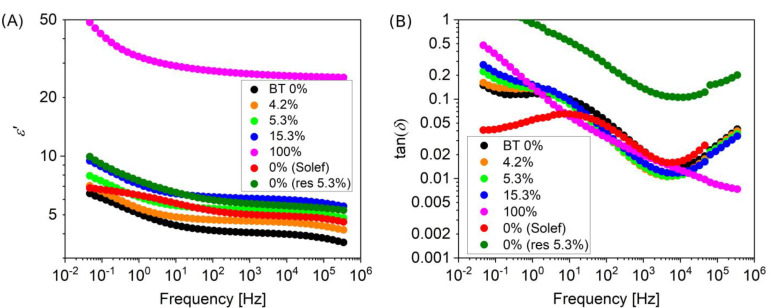
Frequency dependence of the dielectric constant (**A**) and loss tangent (**B**) of RAFT PVDF, PVDF-*g*-BT nanocomposites with the three different volume fractions of BT, the residual ungrafted PVDF extracted from the 5.3 vol% nanocomposite, the Solef PVDF, and the as-received BT nanoparticles, at 20 °C.

To determine the dielectric constant of the employed commercially available BaTiO_3_ nanoparticles, dielectric measurements of pressed nanoparticle pellets were performed. Figure 2A shows a dielectric isothermal spectrum (at 20 °C) of a pellet of as-received nanoparticles. It is evident how the (effective) dielectric constant *ε*′ of this sample (25–50, decreasing with frequency) results much lower than the one declared by the manufacturer (150). The reduced value of *ε*′ can be ascribed to the fact that the pressed pellet includes air voids among nanoparticles, therefore modifying the effective permittivity. The filling factor of pressed spherical particles should approach 0.74 for the close-packed arrangement. By weighting of the pellet produced by applying a pressure of 1.25 kBar, a 0.73 filling factor has been obtained, therefore very close to the expected one. If the BT pressed nanoparticles are modeled as a continuum structure, and the interstices as disconnected voids, we can attempt to apply the Maxwell Garnett relation [29] that is based on an effective medium approximation, to derive the expected effective dielectric constant, or permittivity, by regarding the material as a nanocomposite made up of a BT matrix (with dielectric constant *ε*_BT_) with inclusions of air (with *ε*_air_ = 1).

For spherical nanoinclusions, the Maxwell Garnett equation reads as [29]:

(2) $$\varepsilon =\varepsilon_{m}\left[1+\frac{3\varphi_{f}\left(\varepsilon_{f-}\varepsilon_{m}\right)}{(1-\varphi_{f})\left(\varepsilon_{f}-\varepsilon_{m}\right)+3\varepsilon_{m}}\right]$$

where *ε* is the effective permittivity of the nanocomposite material, *ε_f_* and *ε_m_* the permittivities of the inclusions (filler) and matrix, and *φ_f_* the volume fraction of filler.

The Maxwell Garnett model assumes spherical inclusions, while the close-packing voids between BT nanoparticles have presumably irregular shape. Furthermore, the presence of water molecules adhering to the surface of nanoparticles cannot be excluded due to their hydrophilic character. Therefore, this model was used just to have a crude estimation of the permittivity of BT, *ε*_BT_, to be used for the subsequent analysis of dielectric data of polymer nanocomposites. Roughly, *ε*_BT_ results about 37.5 using the effective permittivity of the pellet at high frequency that is about 25, and 75.6 using the value at low frequency that is about 50. Values of *ε*_BT_ around 80 were found in the literature [30] on pellets of 150–200 nm BT nanoparticles, produced with the same pressure range (1.0–1.5 kBar), although after sintering at 1300 °C for 1h, a filling factor of 0.84 was obtained, likely due to coalescence of particles. In our case, instead, no sintering or other thermal treatments were performed on the BT pellet, in order not to modify the pristine crystallographic phase of the particles as they were employed to obtain our nanocomposites. Therefore, it is more likely that the BT grains have random orientation of their polar axes, possibly contributing to reduce the effective dielectric constant of the pellet. The same random orientation could be found in the nanocomposites, since no poling procedures were applied during sample preparation that could promote alignment of polar axes of nanoparticles along a common direction. Hence, the same value for the high-frequency dielectric constant, *ε*_∞,BT_, inferred for the packed nanoparticles will be used, for our data analysis reported in the Discussion section, to model the dielectric effect of the particles included in the nanocomposites.

### 3.2. PVDF-g-BT Nanocomposites

Figure 2A exhibits the frequency dependence of the dielectric constant, at 20 °C, for the neat PVDF obtained by RAFT (RAFT PVDF), for the nanocomposites with different fractions of BT filler, as well as for the high molar mass Solef PVDF, for comparison. The increase of the BT amount in nanocomposites results in increased dielectric permittivity, as expected. However, the measured values result higher than the ones derived by simple application of the Maxwell Garnett model [29], suggesting a possible role of interfaces, as discussed in the following.

To be able to explain the effect of filler and matrix on the dielectric permittivity of the nanocomposites, several models have been developed, depending on the typology and concentration of the two phases [29,31,32,33,34]. A simple procedure was proposed here based on accepted dielectric models for the nanocomposite systems, applied under different assumptions case by case as detailed hereafter. Preliminarily, the evaluation of the dielectric behavior of the sole amorphous fraction, *ε*_a_, is needed in order to be able to infer the dielectric behavior of PVDF in nanocomposites with different crystalline fractions. First of all, we consider the neat PVDF sample, and assign to the crystalline form the value *ε*_c_ = *ε*_∞,PVDF_ of the dielectric constant at high frequency, determined by fitting of dielectric data at low temperature (−100 °C), where only the faster relaxation processes can be active, in order to minimize the contribution of all other processes, namely primary relaxations as well as interfacial polarization by free carriers. This value turned out to be *ε*_∞,PVDF_ = 2.59 in our case. We now consider the semicrystalline polymer as a composite dielectric material, with the amorphous fraction as the matrix, and the crystalline fraction as the filler. To calculate the effective permittivity of the PVDF matrix, we use the Bhimasankaram-Suryanarayana-Prasad (BSP) dielectric model [35], more adequate than the Maxwell Garnett model to describe composites with high density of spherical dispersed particles, where their mutual interaction cannot be neglected. The effective dielectric constant of the BSP model reads [35]:

(3) $$\varepsilon =\left[\frac{\varepsilon_{m}\left(1-\varphi_{f}\right)+\left[\frac{3\varepsilon_{m}\varepsilon_{f}\varphi_{f}}{\varepsilon_{f}+2\varepsilon_{m}}\right]\left[1+\frac{3\varphi_{f}\left(\varepsilon_{f}-\varepsilon_{m}\right)}{\varepsilon_{f}+2\varepsilon_{m}}\right]}{\left(1-\varphi_{f}\right)+\left[\frac{3\varepsilon_{m}\varphi_{f}}{\left(\varepsilon_{f}+2\varepsilon_{m}\right)}\right]\left[1+\frac{3\varphi_{f}\left(\varepsilon_{f}-\varepsilon_{m}\right)}{\varepsilon_{f}+2\varepsilon_{m}}\right]}\right],$$

where variables have the same meaning than in Equation (2).

As in the case of the pressed nanoparticle pellet, since the geometry of the crystals composing semicrystalline PVDF cannot be considered to be spherical inclusions, we do not expect very reliable results from the model, so we use its outcome just as a first reference value. As a comparison, the same analysis was applied to the case of the high molar mass PVDF (Solef), as well as to the residual PVDF fraction, obtained by dissolution in acetone of the ungrafted polymer contained in the nanocomposites. Specifically, we have considered the residual fraction from the nanocomposite originally indicated as the 10 wt% BT, that from now on, will be indicated with its actual volume fraction that is the one relevant in modeling, derived after TGA analysis (Table 1), i.e., 5.3 vol%.

The resulting values for the dielectric constant (at 20 °C, 300 Hz frequency) of the amorphous fractions are: 7.07 for RAFT PVDF, 7.93 for Solef PVDF, and 9.66 for the 5.3 vol% residual (Table 1).

The expected permittivity of our PVDF-*g*-BT nanocomposites, indicated as *ε*_t_, was then evaluated by means of the Maxwell Garnett model of Equation (2). BT was considered now as the filler, with its volume fraction from Table 1 and permittivity *ε*_∞,BT_, determined as well from fitting of dielectric data. This system fully complies with the requirements of the used dielectric model, since inclusions are spherical particles with moderate density.

We stress that interfacial contributions are not taken into account by the above models. If the measured dielectric constant, *ε_m_*, results higher than *ε*_t_ derived above, it means that the interface between polymer and BT had the effect to enhance dielectric permittivity, or vice-versa. In the present case, as an example, let us consider permittivity values at 20 °C and the frequency of 300 Hz. With *ε*_∞,BT_ = 23.9 and *ε*_∞,PVDF_ = 2.65, we obtain values of the ratio *ε_m_*/*ε*_t_ of 1.40 for the 4.2 vol% BT composite, 1.31 for the 5.3 vol% composite, and 1.54 for the 15.3 vol% composite. All values are higher than 1, indicating that the interface between PVDF and BT provides an enhancement of the dielectric permittivity, of 40%, 31%, and 54%, respectively. Since at 20 °C the conductivity contribution from free charge carriers has not become dominant yet (resulting as less than 3% for the composite with highest BT fraction, in the conditions of our example, as derived later in Section 3.3), this enhancement seems mainly related to the specific structure of the produced core-shell interface, acting as to enhance the overall dielectric permittivity, regardless the free carrier concentration.

The validity of the conclusions drawn above is based on the assumption that both the crystalline structure (size and shape) and the free charge carriers that contribute to conduction are alike in nanocomposites with different BT fractions. Conduction can be inferred from the dielectric measurements, by including a suitable term in the dielectric fitting function, and this can help to validate our analysis. An estimation of the conductivity contribution to the dielectric constant at the same frequency (300 Hz) and temperature (20 °C) for the three samples provides the following values: 0.019, 0.078 and 0.115 S/m, for increasing BT content (4.2, 5.3, 15.3 vol%), while the one for the neat PVDF amounts to 0.006 S/m. Therefore, the role of BT interfaces seems indeed correlated with the one of conductivity, suggesting the presence of interfacial polarization effects like the MWS one [36]. On the other hand, we characterized neither the size of crystalline inclusions, nor the density of free charge carriers at this stage, therefore we lack the evidence for these quantities to be the same for all samples, and hence, it is not possible to infer whether or not the effect demonstrated in the different nanocomposites is not only due to conductivity but also partly due to the different amount of crystalline/amorphous interface within PVDF in the different samples.

PVDF at the interface with BT nanoparticles is chemically bound, since it is obtained as grafted to the functionalized BT particles. This entails a constraint that could affect the polymer chain dynamics, as shown in the literature [37]. However, there is evidence that residual, ungrafted PVDF is still present in all our nanocomposites, so that not all PVDF can be considered to be constrained. Indeed, it was observed that the adopted procedure for ungrafted polymer extraction, i.e., repeated washing with acetone, was not efficient enough for a complete removal [38]. The higher crystallinity of the purified nanocomposites suggests the remaining ungrafted PVDF being mostly in the crystalline phase.

Table 2 compares the permittivity of BT-*g*-PVDF 5.3 vol% sample with other BT-*g*-polymer nanocomposites [39,40,41,42,43] using controlled radical polymerization such as “grafting from” (RAFT, ATRP) and “grafting onto” process (thiol-ene), leading to chemical bonding between the two phases. Our sample (BT-*g*-PVDF), even with lower wt% of BT in the feed (10%), presents higher permittivity compared to that of BT-*g*-PPFOMA where a fluorinated methacrylate monomer was used, and with higher BT wt% (33%), probably also due to the presence of aggregated BT next to the composite. Using fluorinated olefin (VDF) monomer in our case, and compared to methyl methacrylate (MMA) monomer (using RAFT technique), the permittivity of BT-*g*-PVDF is higher compared to BT-*g*-PMMA, despite the higher wt% of BT in the BT/MMA mixture. This could be attributed to higher polarity of VDF units compared to those of MMA.

### 3.3. Dielectric Relaxation Dynamics

Figure 2B presents the frequency dependence of the dielectric loss tangent (tan *δ* = *ε*″/*ε*′) of neat PVDF and PVDF-*g*-BT nanocomposites, at 20 °C. The curves exhibit two relaxation peaks at around 10^0^ Hz and 10^6^ Hz, named primary (*α*) and secondary (*β*) relaxations, respectively. Similar relaxation processes have been reported in the literature on PVDF as well as on its nanocomposites [44,45,46,47,48,49]. Additionally, in the low frequency part of the dielectric loss, the characteristic contribution of DC conductivity effects can be observed, and attributed to free charge carrier transport either along the specimen, or confined at the interphase between the amorphous polymer and its crystalline fraction, and/or the BT nanoinclusions. For comparison, the dielectric loss of the BT sample is also reported, being relatively featureless apart from the rise at lower frequencies due to conductivity. Such rise is instead much smaller for the Solef PVDF material, demonstrating a smaller conductivity compared to the RAFT PVDF.

**Figure 3:**
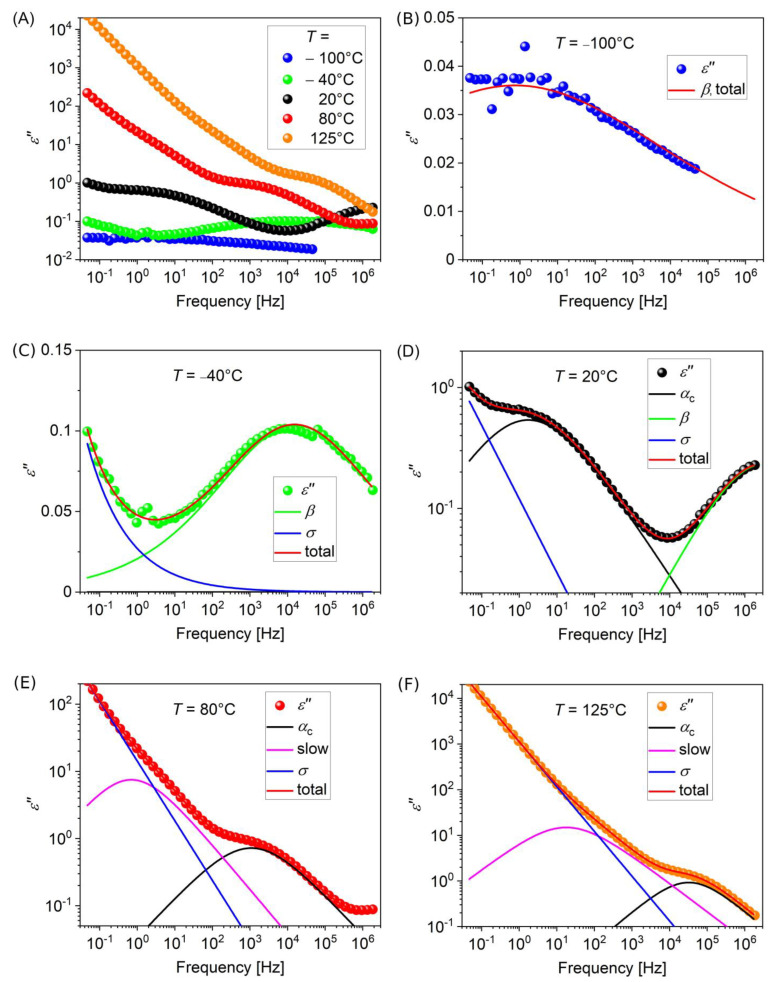
(**A**) Frequency dependence of the dielectric loss (imaginary part of permittivity) of RAFT PVDF at selected temperatures. (**B**–**F**): single spectra at each temperature, along with fitting curves related to the three relaxation processes as well as to the conductivity contribution. Blue curve: conductivity, green curve: *β* relaxation, black curve: *α*_c_ relaxation, magenta curve: slow relaxation, red curve: overall fitting.

Selected spectra showing the frequency dependence of dielectric permittivity for PVDF at different selected temperatures are shown in Figure 3 (the complete spectral sets are reported in Figure S2 of the Supplementary Materials). In addition to primary and secondary relaxations related to PVDF, interfacial or Maxwell-Wagner-Sillars (MWS) polarization can also be observed in dielectric spectroscopy of nanocomposites [50]. In the present case, our estimations suggest indeed the presence of such a mechanism at lower frequencies. Therefore, we have considered in our analysis the presence of a third relaxation process, referred to as “slow”, to account for this aspect. Such a “slow” process was previously observed in the literature [45,51] as arising after introduction of inorganic nanoinclusions in amorphous polymers, whereas the neat polymer showed no such effect. This process can be expected to be due to interfacial polarization by free charge carriers at the interface with nanoinclusions. In the case of semicrystalline polymers, it may also concern polarization at the interface between crystalline domains and surrounding amorphous polymer, as actually observed in our case also for neat PVDF, as well as in the literature [45].

To analyze quantitatively the occurring dielectric relaxations, Havriliak-Negami (HN) functions were used here. In addition, a conductivity term was also included, to take into account the previously mentioned electrode and interfacial polarization. Therefore, the model dielectric function used for our fittings was:

(4) $$\varepsilon^{*}\left(f\right)=\varepsilon_{\infty }+\Sigma_{k}[\frac{\Delta \varepsilon_{k}}{{\left(1+{\left(i\frac{f}{f_{0k}}\right)}^{a_{k}}\right)}^{b_{k}}}]+\frac{\sigma_{0}}{\varepsilon_{0}{\left(i2\pi f\right)}^{n}}$$

where Δ*ε_k_* is the dielectric relaxation strength of the *k*-th process (that we named *α*, *β*, and slow relaxations), *ε*_∞_ is the high-frequency limit of the dielectric permittivity, *f*_0*k*_ is the relaxation frequency of the *k*-th process, *a_k_* and *b_k_* are the parameters describing symmetric and asymmetric widths of the distribution of relaxation times of the *k*-th process, respectively, *σ*_0_ the direct-current conductivity, and *n* a conductivity fractional exponent (0 < *n* < 1) that can describe different conduction mechanisms [52]. In our analysis, the imaginary part of Equation (4), i.e., the dielectric loss term, was used for fitting of *ε*″ experimental data.

Examples of fitting curves for PVDF at different temperatures are reported in Figure 3B–F. Relaxation spectra of all samples were fitted using the same fit constraints, as follows. *β*-relaxation was always assumed as symmetric (*b_β_* = 1), and included in the fitting function up to 25 °C; for higher temperatures up to 90 °C, this process was still included in the fitting, but with a constrained peak frequency obtained by an extrapolation of the *β*-relaxation peak frequencies at lower temperatures by an Arrhenius law. These constrained points were not reported in the relaxation plots (shown in the following), since they were not obtained by direct fitting, but they were only included to improve fitting of the concurrent processes. *α*-relaxation was included, instead, for temperatures higher than −15 °C, while the slow process was included for temperatures higher than 45 °C. Finally, the conductivity exponent had often to be constrained to *n* = 0.4 for temperatures lower than 5 °C, in order to obtain reasonable results for both *α* and *β* relaxations.

**Figure 4:**
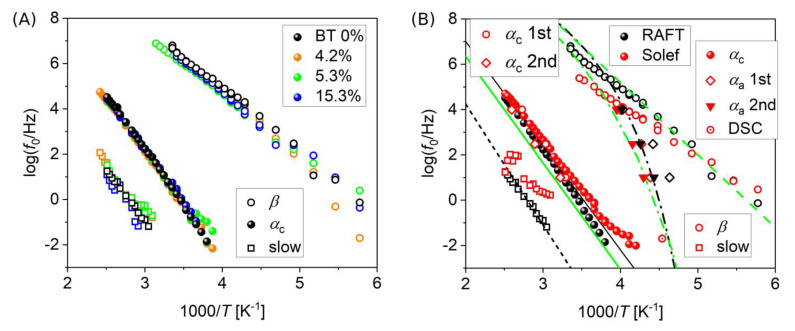
Relaxation plots for different PVDF samples, resulting from fitting of dielectric loss spectra. (**A**) Comparison among nanocomposites with different BT contents. (**B**) Comparison between PVDF obtained by RAFT polymerization (RAFT, black symbols) and commercial, high molar mass PVDF (Solef, red symbols). Lines indicate literature data from two different studies (black lines from Ref. [45] and green lines from Ref. [56]), for all the reported processes: dashed line for the *β* process, dash-dot lines for the *α*_a_ process, solid lines for the *α*_c_ process, dotted line for the “interfacial” process of Ref. [45] (corresponding to our slow process). Three points derived from our BDS isochronal spectra (as the ones shown in Figure 5 below) are also reported for both materials, pertaining to both the *α*_a_ and the *α*_c_ processes, on the 1st heating (*α*_a_, *α*_c_ 1st), as well as on the 2nd heating (*α*_a_, *α*_c_ 2nd), after cooling with the same rate of 10 °C/min. One point corresponding to the calorimetric glass transition temperature derived from DSC thermograms for Solef PVDF (shown in Figure 6D below) is also reported.

Figure 4 shows relaxation plots describing all the observed processes. The logarithm of the relaxation frequency *f*_0_ is reported in an Arrhenius representation, i.e., as a function of inverse temperature (1/*T*). Figure 4A shows the comparison among the neat RAFT PVDF sample and all the related nanocomposite samples, while Figure 4B shows the comparison with the Solef PVDF. It is observed that both the observed primary (*α*) and secondary (*β*) relaxations exhibit a simply activated behavior that is described by the Arrhenius equation [53]:

(5) $$f_{0}=f_{\infty }\exp\left(-\frac{E_{a}}{k_{B}T}\right)$$

where f_∞_ is the relaxation frequency at infinite temperature, *E_a_* the activation energy, and *k*_B_ the Boltzmann’s constant (1.38 × 10^−23^ J/K). This is consistent with previous studies [44,45], in which the two processes were assigned as the relaxation of the crystalline part (*α*_c_) and the *β* relaxation.

The *α*_c_ relaxation is known to arise from dipolar reorientations in the PVDF crystalline region. Its molecular origin has been attributed in the literature to motions of portions of macromolecular chains within the crystalline region, allowed by different types of imperfections and defects of the crystalline packing, or at the lamellar surface [45,46,54,55]. Instead, the *β* relaxation is usually related to local reorientational motions of molecular dipoles [44,56]. Fitting results for both *f*_∞_ and *E_a_* for the three relaxation processes identified in isothermal spectra are reported in Table 3.

**Figure 5:**
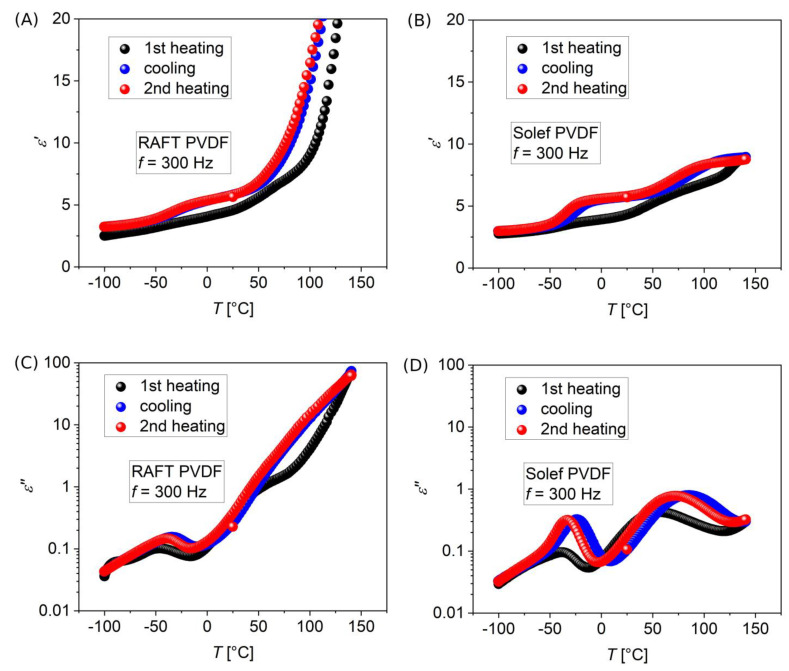
Isochronal dielectric spectra showing the dielectric permittivity (real part *ε*′ (**A**,**B**) and imaginary part *ε*″ (**C**,**D**)) at the frequency of 300 Hz during a temperature ramp from −100 °C to 140 °C (black circles, first heating, at 10 °C/min), successive cooling down to −100 °C at the same rate (blue circles), and second heating up to 140 °C at the same rate (red circles). (**A**,**C**) PVDF sample obtained by RAFT polymerization (RAFT); (**B**,**D**) Commercial high molar mass PVDF (Solef). Peak values of *ε*″ of the lower temperature process, around *T*_g_, reported in the relaxation plot of Figure 4, were derived from (**C**,**D**) as well as from the similar measurements at 10 Hz and 10 kHz (reported in Figure S3 of the Supplementary Materials).

**Figure 6:**
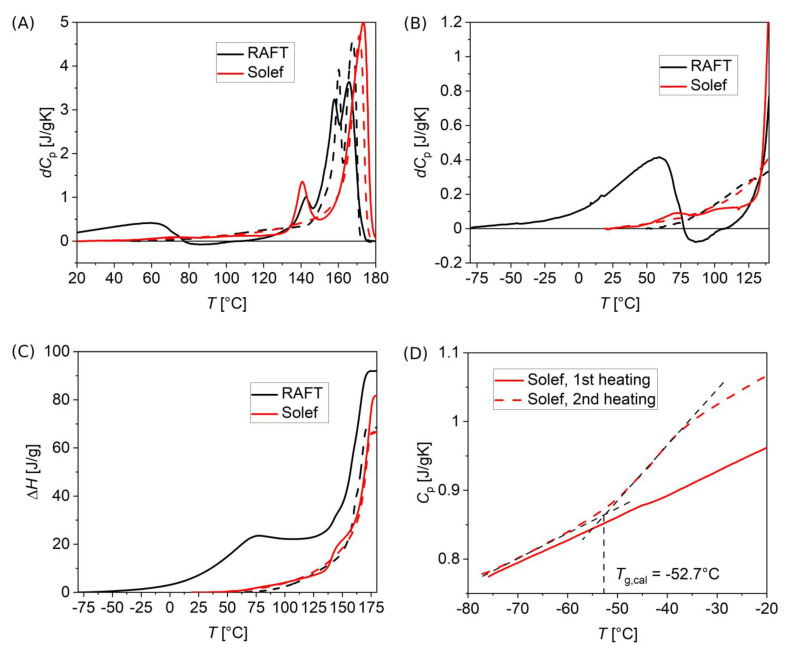
DSC thermograms of PVDF samples obtained by RAFT polymerization (RAFT, black curves) and commercial high molar mass PVDF (Solef, red curves), by ramping temperature starting from −80 °C to 180 °C at a rate of 10 °C/min, for the as-produced samples (solid curves), and after successive cooling (dashed curves). (**A**) Specific heat overview, after a baseline subtraction (indicated as *dC*_p_), showing all melting processes. (**B**) Specific heat detail in a temperature range comparable to that of BDS isochronal scans of Figure 5. (**C**) Specific enthalpy, in the full temperature range. The measurements on nanocomposites (Figure S1 of the Supplementary Materials) showed no qualitative difference with respect to the neat RAFT polymer. (**D**) Determination of the calorimetric glass transition temperature *T*_g,cal_ of Solef PVDF, from the change in specific heat *C*_p_, better evincible from the 2nd heating ramp, after cooling at the rate of 20 °C/min.

### 3.4. Phase Properties of As-Produced RAFT PVDF

To better characterize the glass transition as well as the thermal properties of crystal phases present in our as-produced RAFT PVDF, isochronal BDS complemented by DSC thermal analysis were applied. Indeed, the literature [44,45,56] reports an additional relaxation process, with intermediate timescale, and with a Vogel-Fulcher-Tammann (VFT) temperature dependence, attributed to the structural relaxation of the amorphous phase (*α*_a_ in the cited literature), responsible for the process of glass transition, occurring around T_g_~−40 °C in PVDF [6]. It should be stressed that this process turns out to merge with the *β* relaxation at higher temperature, on the timescale of μs. We have not been able to observe such an *α*_a_ process in isothermal BDS spectra, likely because of the low amorphous content of our samples. Indeed, a low dielectric strength for this process is expected, leading to small contribution in dielectric losses with respect to the concurrent processes in Equation (4). Additionally, all processes are rather broad in our case, increasing their possibility to overlap. Indeed, the symmetrical broadening exponent *a* of Equation (4) ranges between 0.4 and 0.7 for the primary relaxation, and from 0.2 to 0.7 for the secondary relaxation. Refined analysis methods are available in the literature to perform more detailed analysis in similar cases [57]. One of the possible strategies that we have adopted, is the performance of isochronal dielectric spectroscopy. Indeed, in our case there is no temperature range where the different relaxation processes appear as separated enough to facilitate their identification in isothermal spectra. However, since both activation energy and temperature dependence of the dielectric strength of the processes at hand are rather different from each other, it is possible to evidence such different processes by ramping the temperature at a fixed frequency.

To characterize possible peculiarities of PVDF produced by RAFT polymerization method, as for instance the properties of its crystalline structure, investigation was focused on as-prepared samples. Only first heating ramps should be considered, since after heating of the material, some of the crystalline phase could be melted, and recrystallization could happen in a different phase or with a different yield, depending on the reached temperatures and on conditions of the subsequent cooling and re-heating. In view of a possible comparison with DSC thermograms, rates of temperature ramps were adopted to match the typical DSC ones, specifically 10 °C/min.

In Figure 5, heating-cooling-re-heating ramps are shown for PVDF obtained by RAFT polymerization as well as for the commercial, high molar mass PVDF already used for a comparison of isothermal spectra previously shown. We observe a higher value of ε′ on as-prepared PVDF at room temperature, compared to the commercial one (4.45 instead of 4.23, at 20 °C and at 300 Hz frequency). On successive cooling though, the dielectric permittivities result more similar to each other (5.73 instead of 5.77). By performing the second heating, substantial reproducibility with respect to the preceding cooling was found for both polymers, apart from the possible effect of mechanical deformation of the pellets in the BDS measurement cell during thermal cycling. Finally, the strong rise of ε′ at high temperature of RAFT PVDF is assigned to its much higher electrical conductivity compared to the commercially available (Solef) PVDF.

To elucidate the phase and structural transitions of our materials, DSC thermograms for the same samples were taken, shown in Figure 6. For the as-produced RAFT PVDF, a pronounced melting peak at low temperatures (from −80 °C to 60 °C) compared to the main melting (starting at about 150 °C) is visible on first heating (solid black curves). Such a peak is not present after cooling from the melt (Figure 6A, dashed black curves). The same low-temperature melting is not obvious for the nanocomposites (Figure S1 of the Supplementary Materials), while it is more noticeable in the sample composed by the residual ungrafted polymer (Figure S4 of the Supplementary Materials), although with a less pronounced amount. The same effect was not observed on the Solef PVDF (Figure 6A, red curves). Tentative interpretation of our results will be provided in the following Discussion section.

## 4. Discussion

As is well-known, addition of nanoparticles with high dielectric permittivity to a polymer matrix is an effective method to increase the dielectric constant of the nanocomposite. In the present case, this is the main effect used, aimed to increase storage capacity of the material.

The particular interface present in our materials appears to improve of about 50% the dielectric permittivity of the nanocomposite when compared to what expected for a material with the same geometry and composition, but with no specific role of the interface. This can be inferred by comparing the measured dielectric constant to the predicted values by the Maxwell Garnett dielectric model, when considering the nanocomposite material obtained assuming our BT nanoparticles as the filler, and our PVDF as the matrix.

From combined dielectric and thermal characterization, the relative amounts of amorphous and crystalline fractions can be estimated. Our as-produced polymers, and their related nanocomposites, show lower dielectric constant compared to the same materials after a melting-cooling sequence (Figure 5), where recrystallization happens to a lesser extent than with the original production route, thus indicating smaller amorphous fractions. This can be evinced also from calorimetric data of Figure 6 that yield a smaller specific melting enthalpy on the second heating ramp (Figure 6C). Additionally, the difference in specific heat related to glass transition is increased after the first melting and the following cooling (Figure 6D). Since the glass transition is related to the amorphous material, this increase is consistent with the smaller crystallinity obtained after first melting and cooling ramps.

Analysis of thermograms of Figure 6 provides information on the phase properties of materials produced by RAFT polymerization. The main endotherms associated with melting are located in the temperature range between 155 °C and 175 °C, in agreement with the literature (Figure 6A) [5]. A double endotherm is found for the RAFT PVDF, both on the first and second heating, that is commonly observed due to melting and reorganization of crystals when ramping temperature at low rates [58]. Such a double endotherm is instead absent in the high molar mass Solef PVDF, suggesting some role of the chain terminations in crystal reorganization, since they are many more in the much lower molar mass RAFT PVDF. An additional small peak at 140 °C is found for both RAFT and Solef PVDF on the first heating only, not found in the literature. Additionally, a wide peak, between −80 °C and 60 °C, is observed on first heating in RAFT PVDF only (Figure 6B) that deserves special attention. Such an endotherm could be due either to relaxation from some nonequilibrium state pertaining to the glass, due to the long annealing occurred at 60 °C, performed to remove the residual solvent after production, or to melting of a peculiar crystalline phase with low melting temperature compared to the more stable α, β, γ phases. The broadness of the observed peak suggests the first scenario, although the large magnitude of the excess enthalpy, combined with the observed inversion of heat flow after its increase (negative *dC*_p_ between 60 °C and 100 °C in Figure 6B), leads to the second one.

We just remark here that this behavior is peculiar of the samples obtained by the RAFT procedure, since the same effect is not observed on the high molar mass Solef PVDF. This effect, however, appears as strongly reduced in nanocomposites, while it is still present, although less pronounced, in the residual ungrafted polymer sample (Figure S4 of the Supplementary Materials). Since both neat and BT-added PVDF were obtained by the same procedure, the only difference being that the ungrafted polymer was removed from the nanocomposites by washing with acetone, we can draw the conclusion that the low-temperature excess enthalpy was mainly related to the polymer portion that was possible to be removed by washing. On the other hand, after removal by solvation and subsequent reformation of the solid polymer material after annealing (in the PVDF-residual sample), the structure of the ungrafted polymer could be different from the one of the as-produced polymer, since after solvent removal, crystallization may happen in a different manner. Therefore, we cannot consider the DSC results for the ungrafted fraction as conclusive evidence for our analysis.

Finally, as shown in Figure 4B, the *α*_a_ relaxation process, demonstrated by means of isochronal BDS, has a VFT temperature dependence, in agreement with what is reported in the literature for the *α*_a_ process (dash-dot lines in Figure 4B) and with the calorimetric *T*_g_ value obtained by DSC, showing an overall consistency of the obtained information.

## 5. Conclusions

The dielectric properties of core-shell structured nanocomposites made by PVDF-*g*-BaTiO_3_ nanoparticles were investigated. The dielectric permittivity of the nanocomposites increased from the value of the neat PVDF after adding only 15.3 vol% of BaTiO_3_ to the polymer matrix, providing an enhancement of permittivity (at 20 °C) ~50% higher than predicted by the application of the Maxwell Garnett dielectric model to the nanocomposite. This confirmed the advantageous role of the PVDF/BT interface for the performance of core-shell nanocomposites produced here by chemical grafting-from process as materials for application to energy storage. Dielectric relaxation processes investigated by Broadband Dielectric Spectroscopy are consistent with the ones reported in the literature, and showed no significant changes after addition of nanoparticles, probably due to the reduced filler fraction of the studied samples (15.3 vol% maximum). Pronounced ionic conductivity was present in the analyzed samples, showing up with a strong interfacial polarization contribution, compared to a commercial, high molar mass PVDF analyzed for comparison. Finally, an unexpected excess enthalpy at low temperatures compared to the melting of PVDF crystalline phases was detected in the neat RAFT PVDF sample. It could be speculated that such excess enthalpy could be a peculiarity of the most soluble fraction of this RAFT-produced polymer, the nature of which should be further investigated.

Future directions of this research are toward improvement of dielectric breakdown and permittivity based on the devised materials. We performed preliminary tests of dielectric breakdown on our pellets that were previously used for BDS characterizations, because of shortage of materials. Since the used pellets underwent a thermal cycle from −100 °C to 125 °C for BDS isothermal spectroscopy, the tested specimens could have a different structure than the pristine material. Indeed, from DSC characterization, melting and recrystallization of some crystalline phase of the neat RAFT PVDF occurs already below 125 °C, while it is not the case for the polymer within the nanocomposites. In addition, the available pellets were too thick (200 to 500 μm) to achieve reliable dielectric breakdown tests. On the available samples, worsening of the dielectric breakdown was detected for the nanocomposites compared to the neat polymer. Nevertheless, investigation of pristine materials, fabricated in thinner pellets (<100 μm), should be performed in order to obtain more reliable results on the dielectric breakdown of the as-produced materials. This was planned for future work.

The present insulating core-shell PVDF-*g*-BaTiO_3_ 15.3 vol% nanocomposite with improved dielectric permittivity will be used as the filler with a commercially available fluorinated copolymer matrix such as poly(VDF-HFP) using solution blending to develop core double shell structured nanocomposites PVDF-*g*-BaTiO_3_@poly(VDF-HFP). The merit of this method is that the insulating fluoropolymer shells (PVDF-*g*-BT) have similar chemical structure and surface energy with the poly(VDF-HFP) matrix, which not only could enhance the dispersion of BT nanoparticles, but also could improve the interfacial adhesion between nanoparticles and fluoropolymer matrix in comparison to non-fluorinated modified BaTiO_3_ nanoparticles, and therefore improve the dielectric permittivity of the poly(VDF-HFP) matrix. This study is currently in progress.

## Figures and Tables

**Table 1:** Weight loss at 700 °C, specific melting enthalpy Δ*H*_m_, and derived crystalline fraction *X*, for the different PVDF-*g*-BT nanocomposites. Additionally the residual ungrafted polymer obtained by purification of the 10 wt% BT nanocomposite was included.

wt% of BT in Feed	Weight Loss at 700 °C (%) ^(a)^	wt% of BT in Nanocomposite ^(a)^	vol% of BT in Nanocomposite	Δ*H*_m_ (J/g) ^(b)^	Crystallinity *X* (%)
0	100	0	0	83.1	79.5
10 (ungrafted)	==	0	0	82.1	78.6
5	87	13	4.2	87.7	96.4
10	84	16	5.3	78.2	89.0
20	62	38	15.3	63.3	97.7
0 (Solef)	100 ^(c)^	0	0	79.6	76.0

^(a)^ Determined by TGA. ^(b)^ Specific melting enthalpy Δ*H*_m_ measured by DSC analysis under nitrogen atmosphere on first heating, referred to the mass of the PVDF component determined by TGA measurements. ^(c)^ From the Solef^®^ PVDF Design & Processing Guide (Solvay 2017). https://www.solvay.com (accessed July 2022).

**Table 2:** Comparison of the permittivity of BT-*g*-PVDF 5.3 vol% sample with other BT-*g*-polymer reported in the literature.

Core-Shell Structure ^(a)^	wt% BT Amount in Feed ^(b)^	Grafting Technique ^(d)^	Permittivity @1 kHz, RT	Reference
BT-*g*-PPFOMA	33	ATRP	3.5	[39]
BT-*g*-PGMA	25	RAFT	10.0	[40]
BT-*g*-PMMA	25	RAFT	5.0	[40]
BT-*g*-PS ^(c)^	67	Thiol-ene	29.5	[41]
BT-*g*-PMMA	17	ATRP	7.0	[42]
BT-*g*-PS	25	RAFT	24.5	[43]
BT-*g*-PVDF	10	RAFT	5.3	Our work

^(a)^ PPFOMA: poly(perfluorooctylmethacrylate), PGMA: poly(glycidyl methacrylate), PMMA: poly(methyl methacrylate), PS: polystyrene, PVDF: poly(vinylidene fluoride). ^(b)^ m_BT_/(m_BT_ + m_M_) × 100, where m_BT_ and m_M_ are the weights of BT charge and monomer M, respectively. ^(c)^ m_BT_/(m_BT_ + m_PS_) × 100, where m_PS_ stands for the weight of thiol end functional polystyrene PS-SH (1g BT/0.5 g PS-SH). ^(d)^ ATRP: Atom Transfer Radical Polymerization; RAFT: Reversible Addition–Fragmentation chain Transfer.

**Table 3:** Logarithmic relaxation frequency at infinite temperature (log *f*_∞_) and activation energy (*E_a_*) obtained by Arrhenius best fitting of the three observed relaxation processes (as from Figure 4) for RAFT PVDF, PVDF-*g*-BT nanocomposites, residual ungrafted PVDF, and commercially available PVDF (Solef).

Material	Molar Mass [kDa]	Log *f*_∞_ (Slow)	*E*_a_ [kJ/mol] (Slow)	Log *f*_∞_ (*α*_c_)	*E*_a_ [kJ/mol] (*α*_c_)	Log *f*_∞_ (*β*)	*E*_a_ [kJ/mol] (*β*)
PVDF RAFT	22.7	12.62 ± 0.24	86.4 ± 1.7	16.98 ± 0.11	94.2 ± 0.7	16.09 ± 0.18	53.8 ± 0.8
PVDF-*g*-BT 4.2 vol%	14.9	11.90 ± 0.58	80.3 ± 4.2	16.39 ± 0.09	91.9 ± 0.6	17.52 ± 0.28	61.6 ± 1.4
PVDF-*g*-BT 5.3 vol%	13.6	10.11 ± 0.55	67.8 ± 3.7	15.83 ± 0.22	87.2 ± 1.4	15.08 ± 0.12	50.2 ± 0.6
PVDF-*g*-BT 15.3 vol%	12.9	14.20 ± 1.43	99.8 ± 9.7	16.09 ± 0.11	88.9 ± 0.7	15.73 ± 0.19	52.8 ± 0.9
PVDF-res. from 5.3 vol%	13.6	27.62 ± 1.07	185.5 ± 7.5	14.60 ± 0.22	77.1 ± 1.3	14.81 ± 0.17	45.9 ± 0.8
PVDF Solef	352	10.14 ± 1.24	62.4 ± 8.5	15.10 ± 0.18	80.3 ± 1.1	14.78 ± 0.24	50.2 ± 1.2

## Data Availability

The data presented in this study are available in the present article and in the related Supplementary Materials.

## Supplementary Materials

The following supporting information can be downloaded at: https://www.mdpi.com/article/10.3390/polym15030595/s1, Figure S1: DSC thermograms of PVDF-g-BT nanocomposites: 5%, 10%, 20%. q indicates the heating rate; Figure S2: Dielectric loss BDS isothermal spectra of RAFT PVDF; Figure S3: Isochronal dielectric spectra at three different frequencies (10 Hz, 300 Hz, 10 kHz), for the samples investigated in this work: PVDF RAFT, PVDF Solef, PVDF-g-BT 10%, PVDF-g-BT 20%, and residual PVDF extracted from PVDF-g-BT 10%; Figure S4: DSC thermograms of residual PVDF extracted from PVDF-g-BT 10%. q indicates the heating rate.
