# Supplementary material for: Dielectric Characterization of Core-Shell Structured Poly(vinylidene fluoride)-grafted-BaTiO3 Nanocomposites
Source: Polymers (Basel). 2023 Jan 24;15(3):595. doi: 10.3390/polym15030595 (PMC9919282; doi:10.3390/polym15030595)
Supplement: Supplementary file 1 [file polymers-15-00595-s001.zip › polymers-2115596-supplementary.pdf]

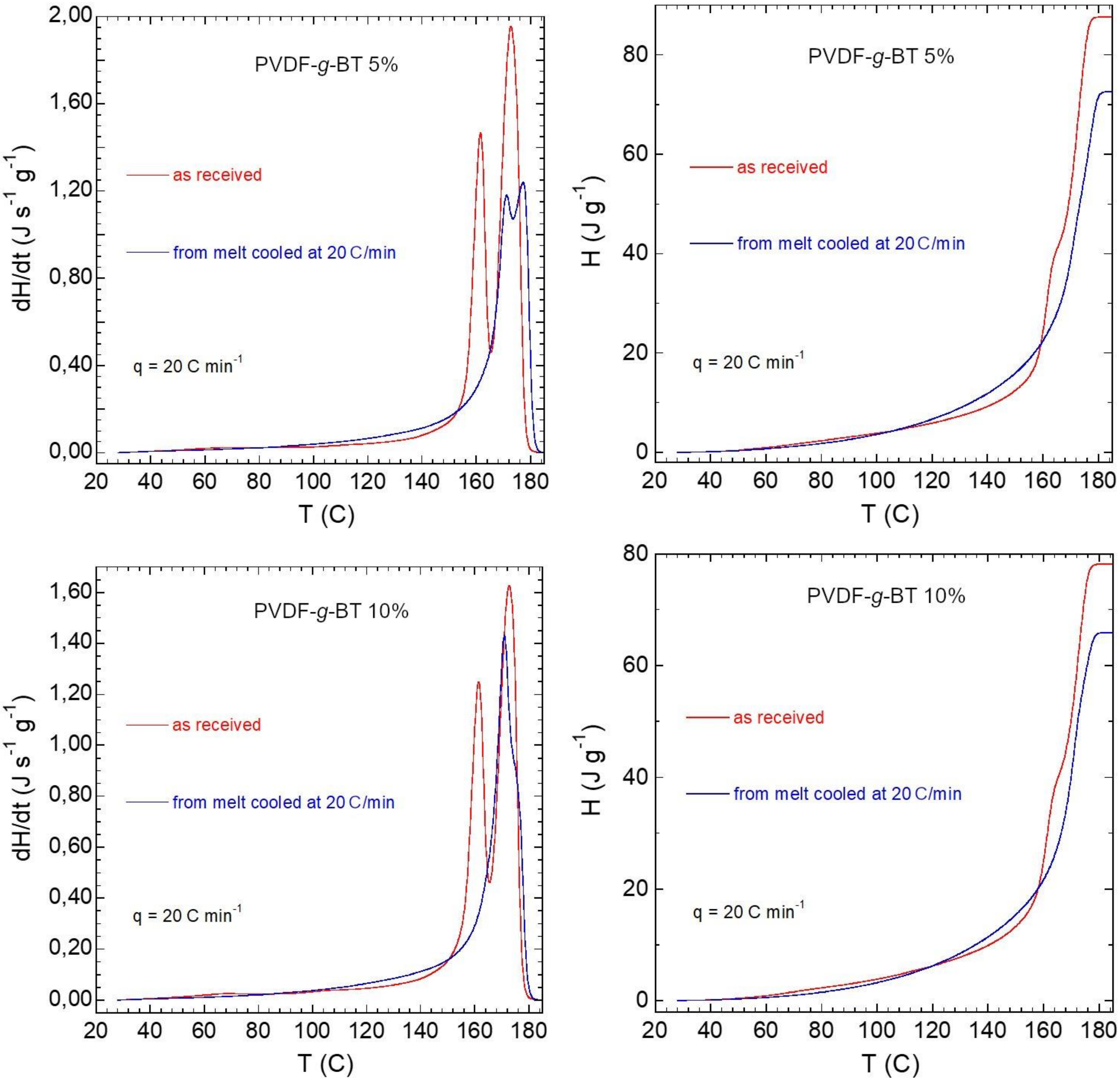
PVDF-g-BT 5%
as received
from melt cooled at 20 C/min
q = 20 C min-1
dH/dt (J s-1 g-1)
T (C)
H (J g-1)
PVDF-g-BT 10%

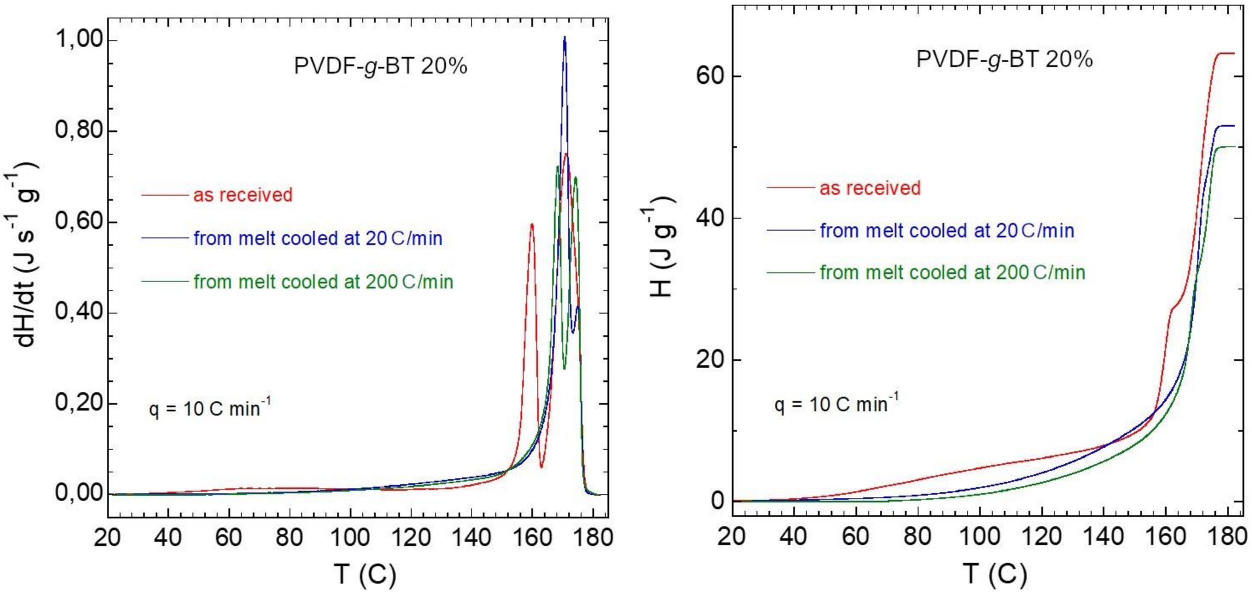

Figure S1. DSC thermograms of PVDF-*g*-BT nanocomposites: 5%, 10%, 20%. q indicates the heating rate.

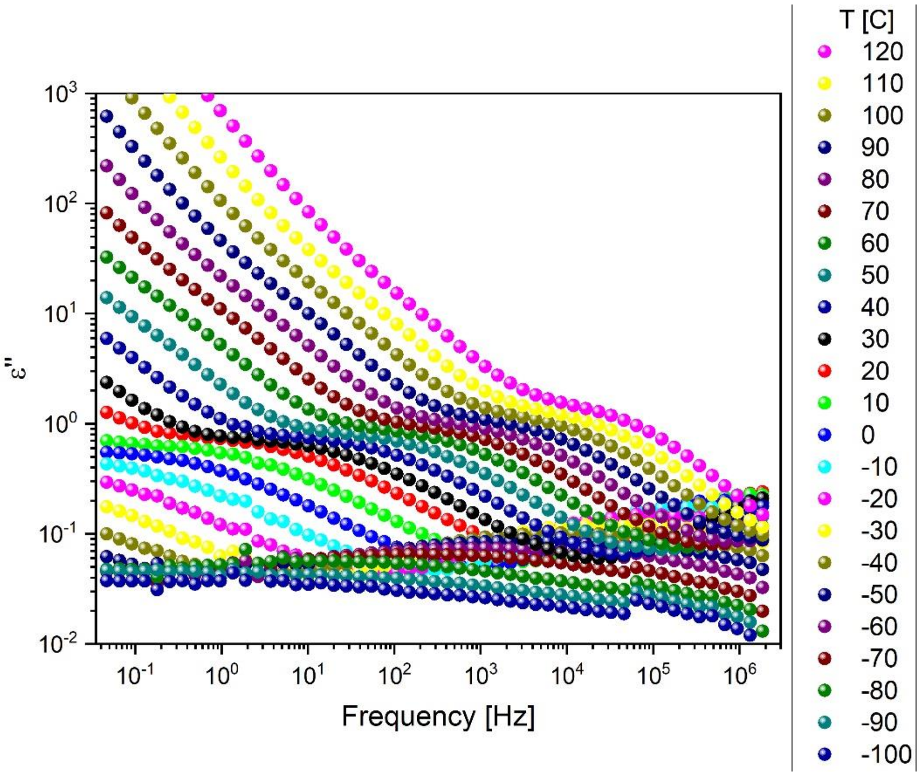

Figure S2. Dielectric loss BDS isothermal spectra of RAFT PVDF.

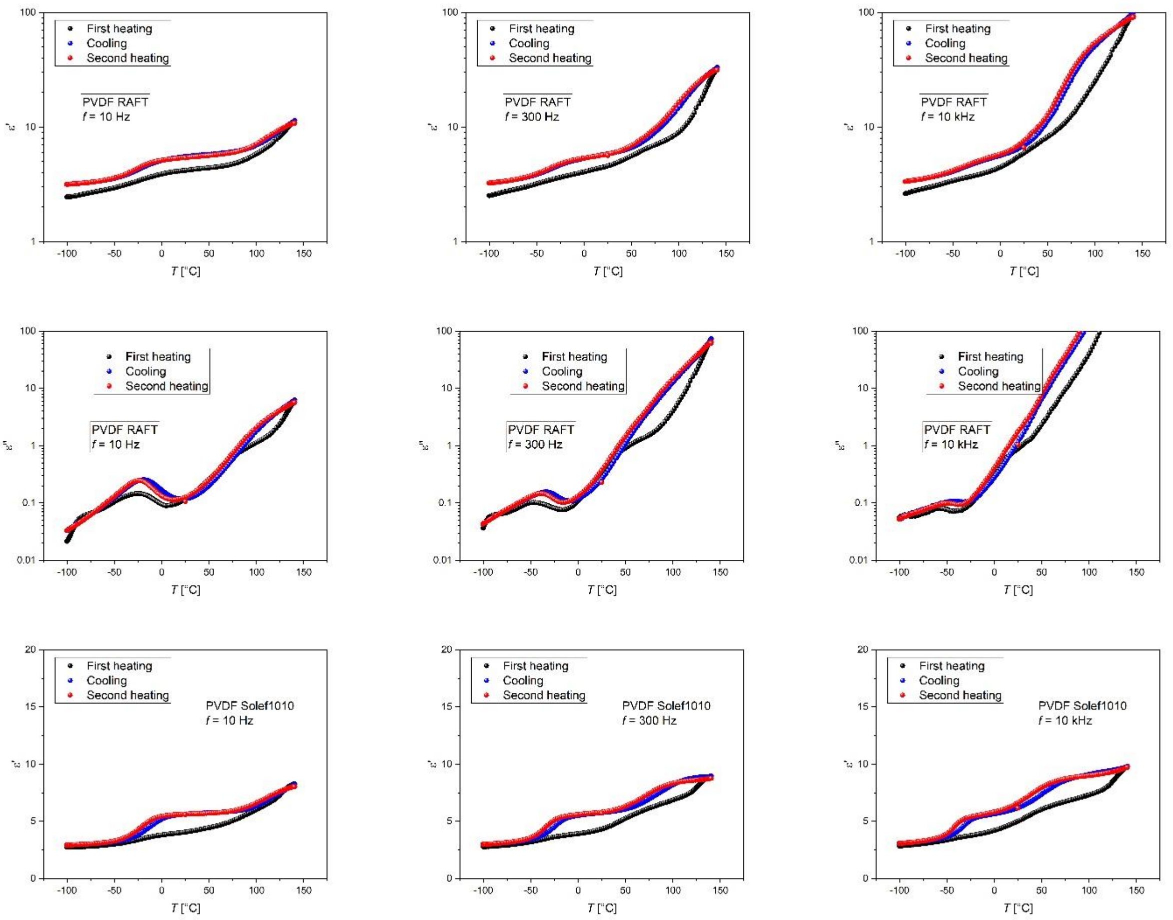

First heating
Cooling
Second heating
PVDF RAFT
f = 10 Hz
f = 300 Hz
f = 10 kHz
PVDF Solef1010
ε'
ε''
T [°C]

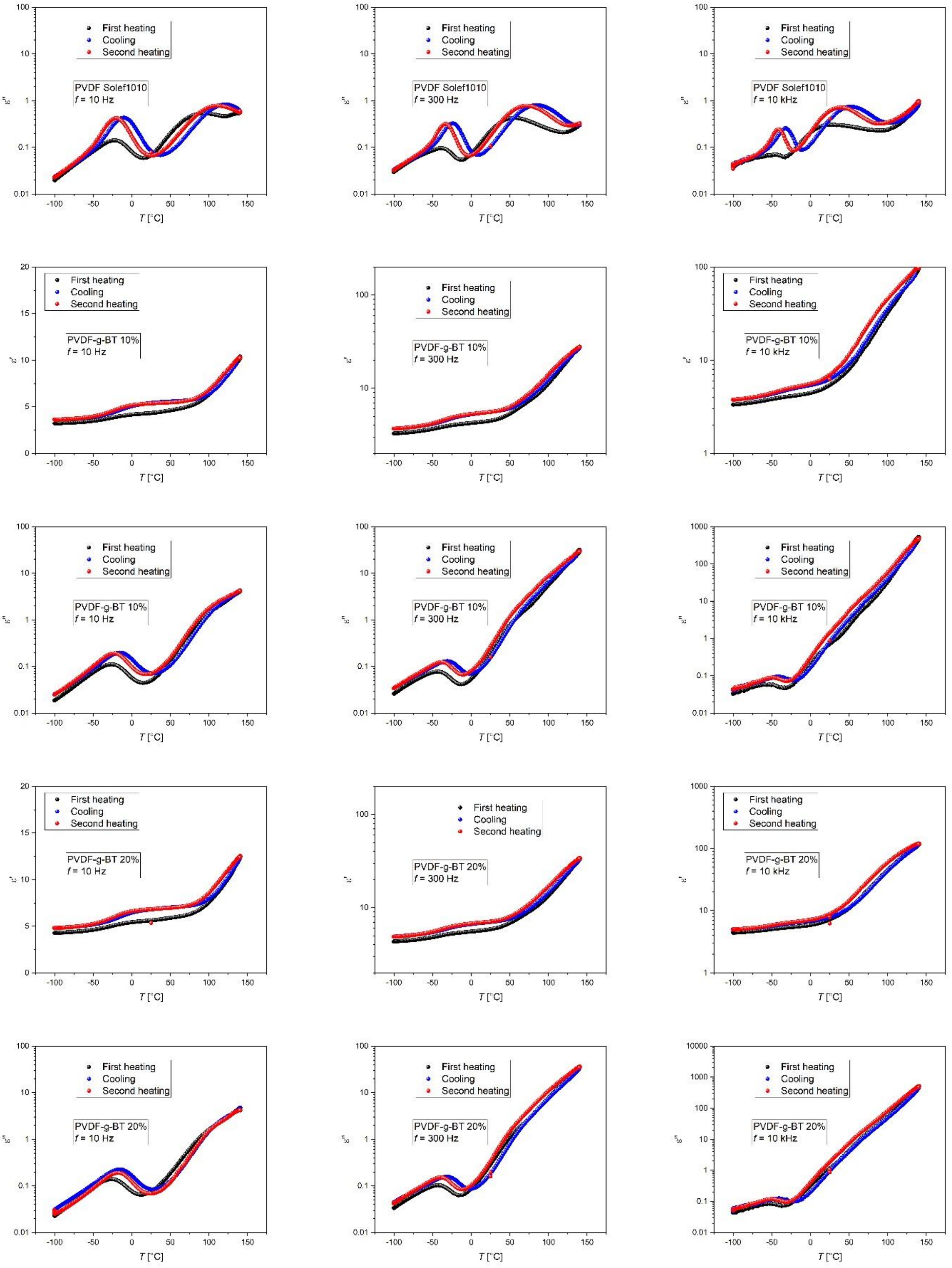

First heating
Cooling
Second heating
PVDF Solef1010
f = 10 Hz
f = 300 Hz
f = 10 kHz
PVDF-g-BT 10%
PVDF-g-BT 20%
ε″
ε′
T [°C]

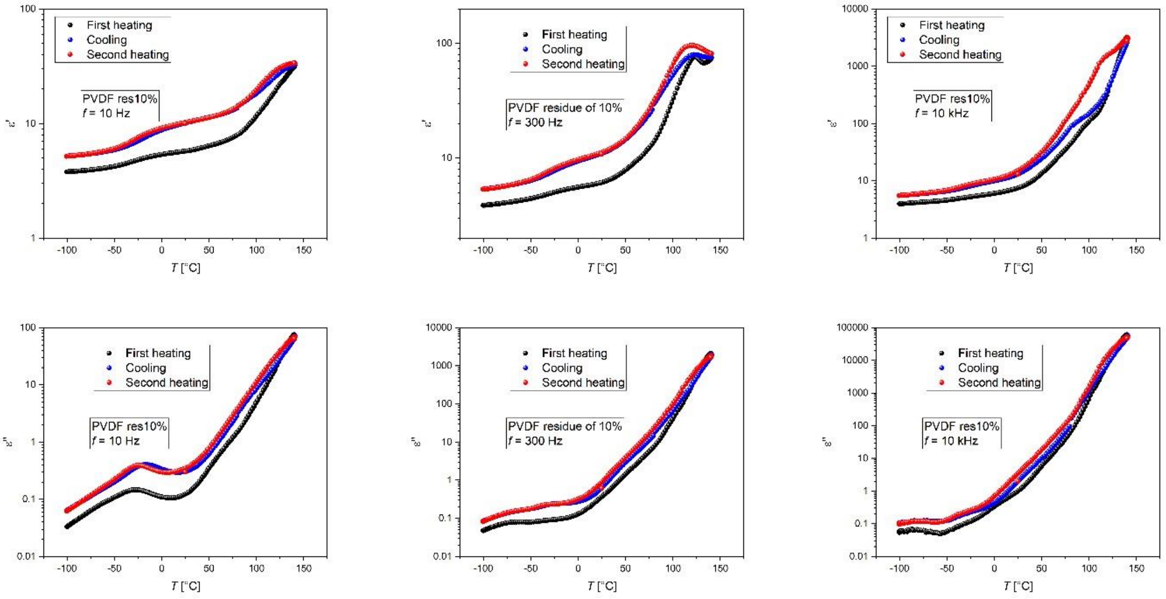

Figure S3. Isochronal dielectric spectra at three different frequencies (10 Hz, 300 Hz, 10 kHz), for the samples investigated in this work: PVDF RAFT, PVDF Solef®, PVDF-*g*-BT 10%, PVDF-*g*-BT 20%, and residual PVDF extracted from PVDF-*g*-BT 10%.

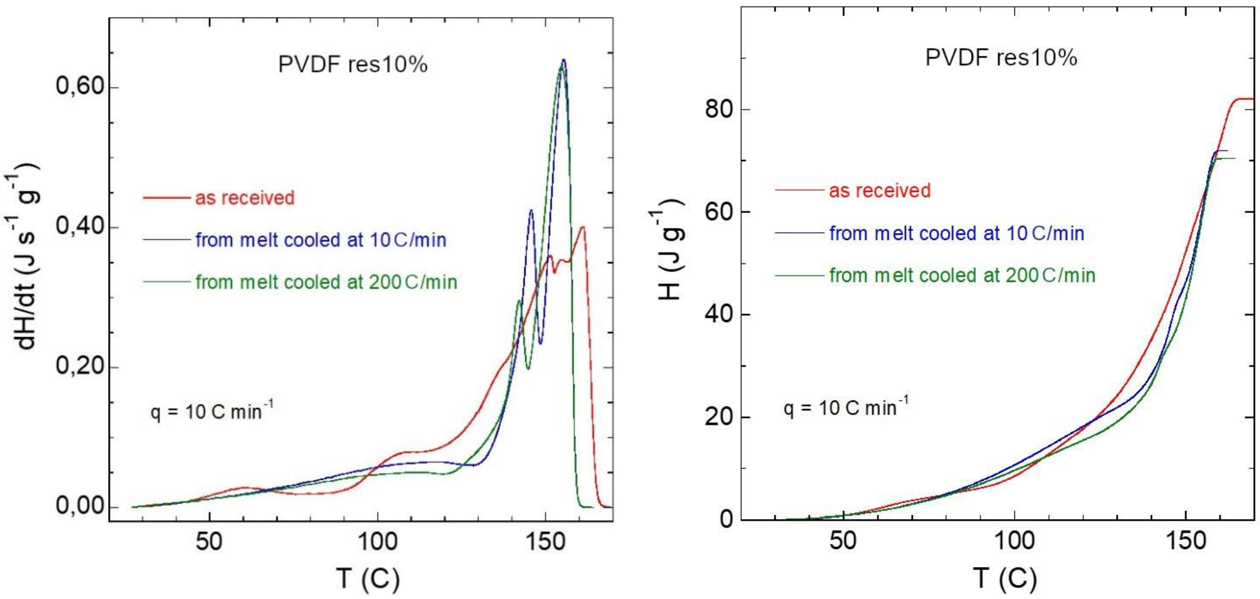

Figure S4. DSC thermograms of residual PVDF extracted from PVDF-*g*-BT 10%. q indicates the heating rate.
